# Genetic Diversity of Vif and Vpr Accessory Proteins in HIV-1 Group M Clades

**DOI:** 10.3390/v18010116

**Published:** 2026-01-15

**Authors:** Oxana Galzitskaya, Aleksey Lebedev, Anastasiia Antonova, Ekaterina Mezhenskaya, Anna Glyakina, Evgeniya Deryusheva, Ilya Likhachev, Anna Kuznetsova

**Affiliations:** 1 Gamaleya National Research Center for Epidemiology and Microbiology, 123098 Moscow, Russia; ogalzit@vega.protres.ru (O.G.); anastaseika95@mail.ru (A.A.); belokopytova.01@mail.ru (E.M.); 2 Institute of Theoretical and Experimental Biophysics, Russian Academy of Sciences, 142290 Pushchino, Russia; 3 Mechnikov Scientific Research Institute of Vaccines and Serums, 105064 Moscow, Russia; 4 Institute of Mathematical Problems of Biology, Russian Academy of Sciences, Branch of the Keldysh Institute of Applied Mathematics, Russian Academy of Sciences, 142290 Pushchino, Moscow Region, Russia; quark777a@gmail.com (A.G.); ilya_lihachev@mail.ru (I.L.); 5 Institute for Biological Instrumentation, Federal Research Center “Pushchino Scientific Center for Biological Research of the Russian Academy of Sciences”, 142290 Pushchino, Russia; janed1986@ya.ru

**Keywords:** HIV-1, Vif, Vpr, group M, subtypes, diversity, sequences

## Abstract

Vif and Vpr are HIV-1 accessory proteins that create optimal conditions for viral replication. They are considered as potential targets for the development of therapeutic agents. Natural amino acid substitutions in these proteins have previously been associated with disease progression. The aim of this study was to analyze the genetic diversity of Vif and Vpr in HIV-1 group M clades. A total of 5286 sequences were downloaded and analyzed. For 37 clades in group M, the consensus sequences, amino acid natural variation, and clade-specific amino acid residue substitutions (CSSs) were evaluated. Structural analysis and modeling of consensus sequences were performed for subtypes A1, B, C, and D. The average conservation degree in the HIV-1 group M was 86.4% for Vif and 91.3% for Vpr. In both proteins, the lowest amino acid diversity was observed in sub-subtype A6, and the highest in subtype B. In consensus sequences, the substitutions, which might influence pathogenesis, have been determined: in Vif—22H (11_cpx, 91_cpx) and 136P (A6, 01_AE, 15_01B, 59_01B, 89_BF1, 103_01B, 111_01C, 133_A6B), in Vpr—41N (06_cpx) and 55A (B, 07_BC, 35_01D, 56_cpx, 66_cpx, 66_BF1, 71_BF1, 85_BC, 137_0107). In functional motifs, CSSs associated with changes in the chemical properties of amino acid residues were noted. These findings could be taken into account for the development of therapeutic drugs in the future. No correlation was observed between the subtypes and the spatial organization of the oligomeric structures of Vif and Vpr. Using the structural analysis and modeling, it has been shown for the first time that Vif can interact with APOBEC3G as an oligomer.

## 1. Introduction

HIV emerged in the Democratic Republic of the Congo around the 1920s as a result of the zoonotic transmission of the simian immunodeficiency virus to humans. Two types of HIV are known: HIV-1 and HIV-2. HIV-2 spreading remains largely limited to Western part of Africa due to its reduced virulence and infectivity. HIV-1 has been classified into four groups: M, N, O, and P. Group M viruses caused the HIV-infection pandemic, which is characterized by a wide range of genetic diversity and is divided into the following subtypes: A, B, C, D, F, G, H, J, K and L [1,2,3,4]. Genetic differences between HIV-1 subtypes range from 25 to 35%, while within individual subtypes—from 15 to 20% [2]. Over time, the genetic diversity of group M viruses worldwide has been increasing. Regular reclassification for some subtypes is conducted with the identification of additional sub-subtypes [1,3,5]. The number of recombinant forms is constantly increasing. In recent studies, the emergence of new circulating recombinant forms (CRF157_A6C and CRF158_0107) was described [6,7]. Subtype C is the most widespread HIV-1 variant (about 50% of HIV infection cases), subtype A is in second place (about 12%), and subtype B is in third place (about 11%), followed by CRF02_AG (~6.6%) and CRF01_AE (~ 5.4%); for each of subtype G and D—less than 3%; for each of the following variants (URFs, CRF07_BC and other CRFs)—less than 2%; and for each of subtype F, H, J and K—less than 1% [1]. However, HIV variants are spread unevenly around the world: subtype C in Southern Africa and India, Subtype A in parts of East Africa, Russia and former Soviet Union countries, subtype B in Europe, the Americas and Oceania, CRF01_AE in Asia, and CRF02_AG in Western Africa [2]. In the earliest studies on HIV-1 non-B subtypes, it was shown that various HIV-1 variants may have different pathogenesis: subtypes C and D were the most aggressive, followed by G, AE, and AG, herewith A being the least aggressive [8]. Regular study of the characteristics of HIV-1 variants is necessary for diagnosis, treatment, and vaccine development.

Vif and Vpr are HIV-1 accessory proteins that are encoded by adjacent genes, translated from incompletely spliced viral RNA and produced at the late stage of the viral life cycle [9]. The main Vif function is to counteract human cellular restriction factors, the APOBEC3 family of proteins, in a variety of ways: by recruiting them to the ubiquitin ligase complex for subsequent degradation, by hijacking transcriptional co-factor CBF-β, and inhibiting their transcription, translation inhibition and inhibiting their packaging into virions [10,11]. Vif is a highly basic polypeptide consisting of 192 amino acid (aa) residues the primary structure, which contains functional motifs for interaction with members of the APOBEC3 family of proteins and with Cullin 5, Elongin C and CBF-β, subunits of the E3-ubiquitin ligase complex, and also the nuclear transport inhibitory signal [10,12,13]. Vif binds with HIV-1 RNA in the cytoplasm, which, presumably, is related to the chaperone activity of Vif protein, and, in addition, with the necessity of such interaction for Vif incorporation into virions [14,15,16]. Moreover, Vif oligomerization promotes the formation of high-molecular complexes and may be important for activity involving RNA interaction [16,17,18].

Vpr is a multifunctional viral protein which creates optimal conditions for virus replication in various ways: it increases the processivity of reverse transcription, takes part in the nuclear import of viral DNA and enhances viral genome transcription, promotes G2 cell cycle arrest, triggers DNA damage responses and mitochondrial disruption, and prevents Env-positive virions traffic to the lysosome for degradation [19,20,21,22,23,24]. One of the best-known interactions is the opposition of the Vpr to the cellular protein uracil DNA glycosylase 2, UNG2, the overexpression of which results in the disruption of HIV-1 gene transcription through an unknown mechanism [25]. Moreover, Vpr has effects on the CNS, both as an intracellular and an extracellular protein, and leads to the development of neurocognitive disorders [26,27]. Vpr is a highly conserved protein, ~14 kDa, composed of 96 aa residues. Its structure includes three α-helices, located from 17 to 33 aa residues, from 38 to 50 aa residues, from 55 to 77 aa residues and surrounded by flexible N- and C-terminal parts [28]. Vpr oligomerization is a critical process for its interaction with Pr55^Gag^, and subsequently, Vpr incorporation into virions and participation in the early stages of the virus life cycle [29].

The functional activities of Vif and Vpr may vary among HIV-1 variants [30,31,32]. Natural amino acid residues substitutions in Vif and Vpr may affect their functional activities and be associated with differences in the clinical status of patients [33,34,35,36]. Moreover, Vif and Vpr may be considered as targets for the development of therapeutic agents [37,38,39,40]. Therefore, the study of the genetic diversity of Vif and Vpr is of interest. A recent study analyzed the global diversification of Vif in different HIV-1 variants over time [41]. Currently, the molecular genetic characterization of Vif and Vpr in locally circulating HIV-1 variants is under study [31,42,43,44].

The aim of this study was to analyze the genetic variability of Vif and Vpr in HIV-1 group M variants by analyzing sequences available in the Los Alamos National Laboratory HIV sequence database.

## 2. Materials and Methods

### 2.1. Sequence Dataset Compilation

The HIV-1 Vif and Vpr protein complete coding sequences were retrieved from the HIV Sequence Database maintained by the Los Alamos National Laboratory (available at www.hiv.lanl.gov/, accessed on 28 December 2024). The study was restricted to HIV-1 group M sequences; unique recombinant forms (URFs) were excluded from consideration. To ensure data integrity, sequence inclusion was predicated on only near full-length HIV-1 genomic representation, with the subsequent removal of (i) redundant sequences (defined as duplicate sequences originating from the same patient), (ii) sequences exhibiting premature termination codons within the Vif and Vpr coding regions, and (iii) sequences manifesting greater than 5.0% ambiguous amino acid residues. The resultant amino acid sequences underwent alignment against the HXB2 reference strain (GenBank accession number K03455) using the ClustalX algorithm. Post-alignment processing, including analysis and sequence trimming, was performed with MEGA-X software version 10.2.2 [45]. Manual curation of the sequence alignments ultimately yielded a final dataset comprising 5286 Vif and Vpr protein sequences with documented collection dates spanning the period from 1978 to 2023. A comprehensive listing of GenBank accession numbers, geographical origins, and collection dates pertaining to the HIV-1 sequences incorporated in this study is presented in Table S1.

### 2.2. Consensus Sequence Calculation

Consensus sequence calculations were performed employing the Consensus Maker tool (accessible at https://www.hiv.lanl.gov/content/sequence/CONSENSUS/consensus.html, accessed on 28 December 2024), using the entirety of the acquired sequences for each respective HIV-1 clade. To ensure the robustness of the derived consensus sequences, a minimum of three sequences was employed for each HIV-1 clade, facilitating the assignment of the most prevalent amino acid residue to each position without removing the gaps. The Group M consensus sequence was subsequently generated through the integration of all HIV-1 clade consensus sequences.

### 2.3. Amino Acid Frequencies and Diversity

The VESPA tool (accessible at https://www.hiv.lanl.gov/content/sequence/VESPA/vespa.html, accessed on 28 December 2024) was implemented to quantify the frequency of each amino acid residue at each position within the generated sequence alignment. This analytical approach involves the comparative assessment of two distinct sequence cohorts to identify a “signature” pattern, defined as sets of amino acid residues exhibiting conservation within each individual cohort and subsequently quantifying divergent amino acid residues and their respective frequencies within each cohort. This process facilitates the determination of divergent amino acid residues and the quantification of their frequencies within each cohort. The consensus sequence was employed as a background dataset for the intra-cohort analysis. Amino acid residue divergence, quantified as substitutions per site, was calculated for each region utilizing the Jones–Taylor–Thornton (JTT) matrix model and the Gamma model as implemented in MEGA. Pairwise sequence alignments were performed with the exclusion of insertions and deletions present exclusively in individual HIV-1 sequences that were not incorporated within the derived consensus sequence.

### 2.4. Structural Analysis and Modeling

For subtypes A1, B, C and D, and the tertiary structures of dimers, tetramers and hexamers of Vif and Vpr protein sequences, as well as structures of APOBEC3G complexes with Vif, UNG complexes with Vpr were obtained using the AlphaFold3 program (https://alphafoldserver.com, accessed on 6 November 2025) [46]. Tertiary structure alignment and root mean square deviation (RMSD) calculations were performed using the Chimera program (https://www.cgl.ucsf.edu/chimera/, accessed on 6 November 2025). Pairs of contacting residues (contact distance 5Å or less) were calculated for Cα atoms using a script in PyMOL v.2.5.0 (https://pymol.org/2/, accessed on 1 June 2025). The interaction energy of APOBEC3G complexes with Vif, UNG complexes with Vpr were estimated using the MD PUMA-CUDA program, http://lmd.impb.ru/Puma/Manual_PUMA-CUDA.pdf, accessed on 6 November 2025 [47,48].

### 2.5. Calculation of the Variability Index

The protein variability index was determined through the calculation of the Wu–Kabat (WK) variability coefficient for the aligned protein sequences, both within the aggregate dataset encompassing all sequences and within individual HIV-1 clades. The WK coefficient was computed according to the following formula: V_wk_ = N × k/n, where N represents the total number of sequences within the alignment, k represents the number of distinct amino acid residues present at a given position, and n represents the absolute frequency of the most frequently occurring amino acid residue at that position [49]. The Wu–Kabat variability coefficient serves as a well-validated descriptor of the susceptibility of an amino acid residue position to evolutionary substitution, with a WK value exceeding 1 indicative of relative variability at the respective site; specifically, higher WK values correlate with increased diversity at the given position.

### 2.6. Statistical Analysis

This study reports descriptive statistics of amino acid residue changes and the diversity of the Vif and Vpr proteins within individual HIV-1 group M clades. Continuous variables and categorical variables are presented as medians with associated interquartile ranges (IQRs) and as frequencies with corresponding percentages (%), respectively. Pairwise comparisons of amino acid residue diversity and amino acid residue conservation were conducted using the Mann–Whitney U-test and Fisher’s exact test, with application of the Bonferroni correction for multiple test correction (p = α/m, with α= 0.05, m = 1332 tests), respectively. For all statistical tests, a *p*-value of less than 0.05 was considered to denote statistical significance. Statistical analysis was performed using STATISTICA v.10.0 software (StatSoft, Austin, TX, USA).

## 3. Results

A total of 168 HIV-1 clades (subtypes or CRFs) were selected based on the complete Vif and Vpr protein sequences in each set. The number of sequences available for each subtype/CRF is described in Supplementary Table S2. Of those, 37 subtypes/CRFs with more than eight sequences allowed consensus sequences comparison (Table 1).

### 3.1. Amino Acid Residue Variability in HIV-1 Group M Clades

Although amino acid (aa) residue changes were analyzed for each of the 37 HIV-1 clades (Table 1), we restrict our attention here to well-selected clades with more than 50 sequences (A1, A6, B, C, D, F1, G, 01_AE, and 02_AG). Together, these clades revealed a total of 73,284 aa residue substitutions, 476 deletions, and 139 insertions in the Vif protein. Analysis of the Vpr protein showed less diversity in aa residue changes (29,326), but a higher frequency of deletions (526) and insertions (258) (Table 1). Subtype D exhibited the fewest aa residue changes in Vif (11.6 aa residue changes per sequence), while sub-subtype A6 had the fewest changes in Vpr (5.0 aa residue changes per sequence). Subtypes B and G were the most variable (19.6 aa residue changes per sequence for Vif, and 7.5 and 7.4 aa residue changes per sequence for Vpr, respectively). Overall, the proportion of variable aa residue positions in Vif ranged from 52.6% in F1 to 94.3% in subtype B; in Vpr, the proportion of variable aa residue positions ranged from 46.8% in F1 to 97.9% in subtype B (Table 1). The changes in aa residue positions and the percentage of variable positions in individual Vif and Vpr motifs (domains) varied significantly across the HIV-1 group M clades (Table S3). In terms of aa residue diversity in the Vif and Vpr proteins, subtype A6 was the most conserved, with a median of 9.6 × 10^−2^ substitutions/site for Vif and 7.9 × 10^−2^ substitutions/site for Vpr, while subtype B was the most variable, with a median of 17.3 × 10^−2^ substitutions/site for Vif and 12.6 × 10^−2^ substitutions/site for Vpr (Figure S1).

### 3.2. Changes in HIV-1 Consensus Sequences

**Figure 1:**
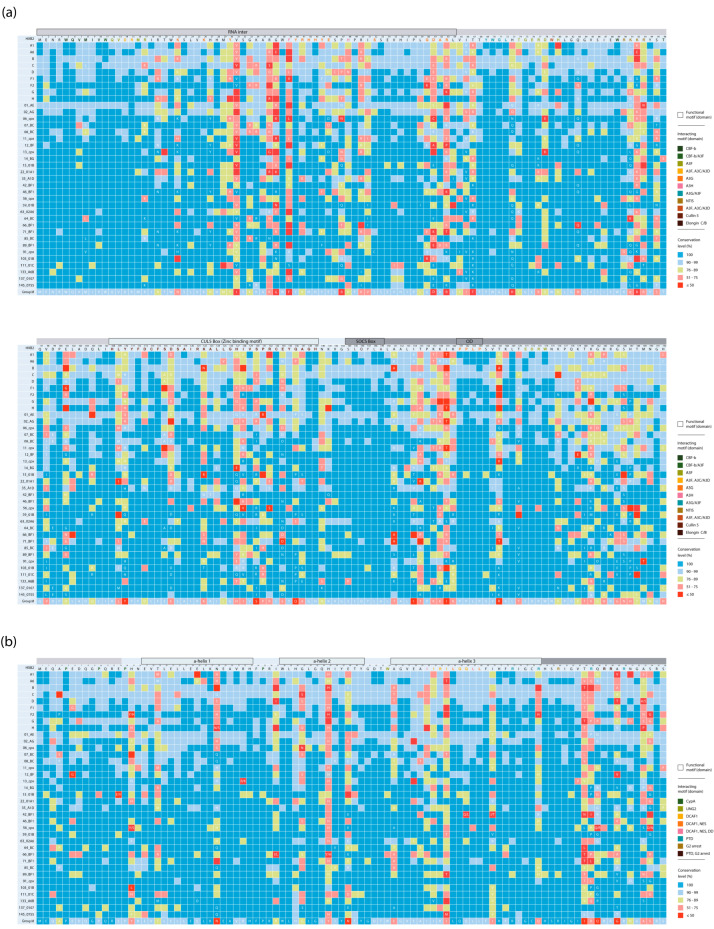
Multiple sequence alignment of the consensus sequences HIV-1 group M clades: Vif (**a**) and Vpr (**b**). Amino acid residues are numbered according to the HXB2 subtype B reference strain presented above the clades. Additionally, consensus sequences were compared to that of the HIV-1 group M consensus presented below the clades. Amino acid residues are colored according to their level of conservation, as indicated in the legend. Empty cells represent the same a.a. as in HIV-1 consensus for that position. The N-terminal regions (N-terms), Vif and Vpr, are marked in gray, and the C-terminal regions (C-terms)—dark gray. Annotated protein motifs (domains) are indicated as rectangles (above the scale): RNA inter, RNA interaction motif, CUL5 Box, Cullin 5 Box (HCCH zinc-binding motif); SOCS Box, suppressor of cytokine-signaling box; OD, oligomerization domain. CBFb, core-binding factor subunit beta; A3(F) (C, D, G, H), APOBEC3(F) (C, D, G, H); NTIS, nuclear transport inhibitory signal; CypA, Cyclophilin A; UNG2, *Uracil-DNA glycosylase 2*; DCAF1, DDB1 and CUL4 associated factor 1; *NES*, nuclear export signal; DD, dimerization domain; PTD, protein transduction domain [10,13,15,16,17,18,28,50,51,52,53].

The HIV-1 group M consensus sequences for Vif and Vpr were constructed based on the consensus of 167 clades. Heterogeneity in consensus sequences was observed at 91 (47.4%) positions in Vif and 29 (30.2%) positions in Vpr between clades, while the remaining sites contained the same aa residues in the consensus sequences, regardless of the level of aa residue conservation (Figure 1). In Vif, the single amino acid residue insertion 141–142insN was found in 6 of the 37 HIV clades: 07_BC, 08_BC, 64_BC, 85_BC, 137_0107, and 145_0755, but this is not shown in Figure 1.

In the Vif protein, the SOCS Box region contained the lowest number of variable positions (16.7%), while the CUL5 Box region had the highest number of variable positions (59.4%); the OD motif contained no variable positions. The N-terminal region had fewer variable positions (43.0%) than the C-terminal region (49.0%). In the Vpr protein, the α-Helix-1 region contained the lowest number of variable positions (23.5%), while α-Helix-2 had the highest number of variable positions (30.7%). The N-terminal region had fewer variable positions (20.1%) than the C-terminal region (47.4%).

**Figure 2:**
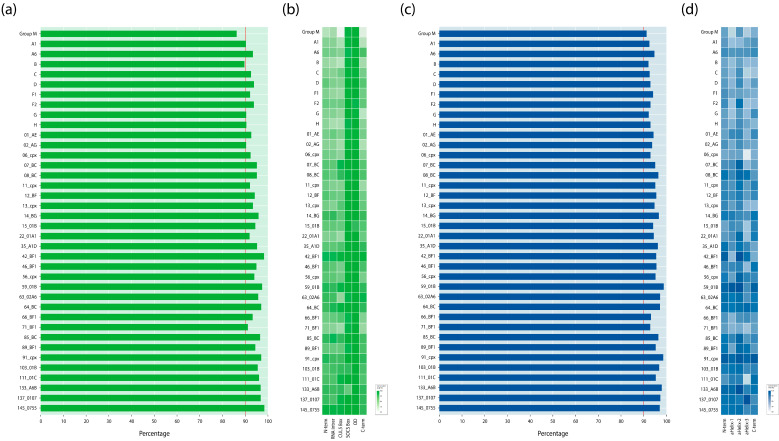
The conservation level of amino acid residues in the full-length Vif and Vpr protein sequences across HIV-1 group M clades. The diagram shows the overall percentage of aa residue conservation in Vif (**a**) and Vpr (**c**) proteins, as well as the percentage of aa residue conservation for each domain of the Vif (**b**) and Vpr (**d**) proteins with the use of a color gradient. Annotated protein motifs (domains) are indicated as follows: N-term, N-terminal region; C-term, C-terminal region; RNA inter, RNA interaction motif; CUL5 Box, Cullin 5 Box (HCCH zinc-binding motif); SOCS Box, suppressor of cytokine-signaling box; OD, oligomerization domain.

The average level of Vif amino acid residue conservation in the HIV-1 group M was 86.4%. Among the well-studied HIV-1 clades with more than 50 available sequences, subtype D showed the highest conservation (94.0%), while subtype B showed the least conservation (89.8%) (Figure 2a).

The most conserved motifs/domains in the HIV-1 group M in Vif were the OD (99.8%) and SOCS Box (99.3%) (Figure 2b). The highest conservation value was 99.9%, found at sites 1, 5, 11, 38, and 53 (RNA inter); 114 and 115 (CUL5 Box); 145, 148, and 149 (SOCS Box); and P162 and P164 (OD).

The least conserved motif was the CUL5 Box (82.5%). The lowest conservation values were found in positions 37 (36.3%, RNA inter) and 110 (39.4%, CUL5 Box). Generally, the conservation patterns in Vif motifs/domains varied slightly between HIV-1 clades (Figure 2b). The range of conservation in individual Vif motifs/domains across different HIV-1 group M clades is described in Supplementary Table S4.

The average level of amino acid residues conservation in Vpr in the HIV-1 group M was 91.3%. The A6 sub-subtype had the highest conservation level (94.8%), while the B subtype—the lowest level (92.2%) (Figure 2c). The most conserved motif/domain in the HIV-1 group M was α-Helix-1 (94.3%) (Figure 2d): 88.2% positions demonstrated greater than 90% level of conservation. The least conserved motif was α-Helix-2 (89.6%): 23.1% positions had less than 90% level of conservation. The highest conservation values (100.0%) were noted in sites 24, 26, 27, 29, 30, and 33 (α-Helix-1), as well as at sites 38, 39, 42, 43, 47 and 50 (α-Helix-2), and 64, 65, 71, 73, 75 and 76 (α-Helix-3). The lowest conservation values were found at sites 28 (41.7%, α-Helix-1) and 48 (48.8%, α-Helix-2). The conservation patterns in Vpr motifs/domains also differed between HIV-1 clades (Figure 2d; Table S4).

### 3.3. Clade-Specific Amino Acid Residue Substitutions in the Vif and Vpr Proteins of HIV-1 Group M

**Figure 3:**
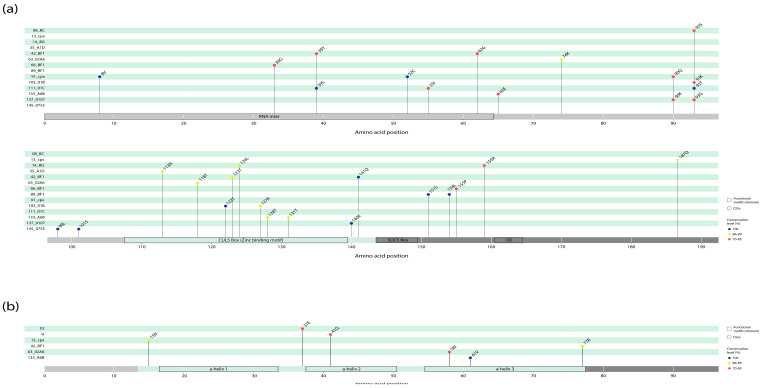
Clade-specific single amino acid residue substitutions (CSSs) in the full-length Vif and Vpr proteins in HIV-1 group M variants. Amino acid residues are numbered according to the reference strain HXB2 subtype B. A total of 38CSSs in the Vif (**a**) and Vpr (**b**) proteins are marked. CSSs are indicated by colors according to the frequency of occurrence. Amino acid residue substitutions relative to the consensus are indicated by single-letter codes. The N-terminal regions (N-terms) in Vif and Vpr are shown in light gray, and the C-terminal regions (C-terms) are shown in dark gray. Annotated protein motifs (domains) are indicated by boxes: RNA inter, RNA interaction motif; CUL5 Box, Cullin 5 Box (HCCH zinc-binding motif); SOCS Box, suppressor of cytokine-signaling box; OD, oligomerization domain.

In Vif and Vpr proteins, 38 CSSs were found in 16/37 HIV-1 clades. The prevalence of CSSs and their locations are shown in Figure 3. CSS changes were more common in the Vif protein (32/38, 84.2%). In Vif, the highest number of CSSs (8 substitutions in six clades) was recorded in the Cul5 Box (zinc-binding) motif protein; in Vpr protein—in the α-Helix-3 motif (three substitutions in three clades). In Vif, the following CSSs had 100% representation: in 91_cpx—8V and 52C, in 111_01C -39S and 93T, in 145_0755—98L and 101S, in 103_01B—122T, in 137_0107—140K, in 42_BF1—141Q, and in 89_BF1—151S and 154L. At the same time, in Vpr, only one CSS had 100% representation: in 133_A6B—61V.

### 3.4. Spatial Alignment of the Monomeric and Oligomeric Structures of Vif and Vpr Modeling of Vif-APOBEC3G and UNG-Vpr Interactions

In Figure S2, the spatial alignments of the dimeric structures of Vif and Vpr proteins for subtypes A1, B, C, and D are presented. The most similar structures for Vif were found between subtypes B and C (RMSD = 3.2 Å), and for Vpr, between subtypes A1 and D (RMSD = 7.6 Å). Figure S3 displays the spatial alignments of the tetrameric structures of Vif and Vpr for subtypes A1, B, C, and D. The most similar structures for Vif occurred between subtypes A1 and C (RMSD = 3.0 Å), while for Vpr—between subtypes B and D (RMSD = 8.2 Å). The spatial alignments of the hexameric structures of Vif and Vpr for subtypes A1, B, C, and D are shown in Figure S4. The most similar structures to Vif were hexamers of subtypes A1 and D (RMSD = 11.870 Å), and for Vpr, hexamers of subtypes A1 and B (RMSD = 21.787 Å).

**Figure 4:**
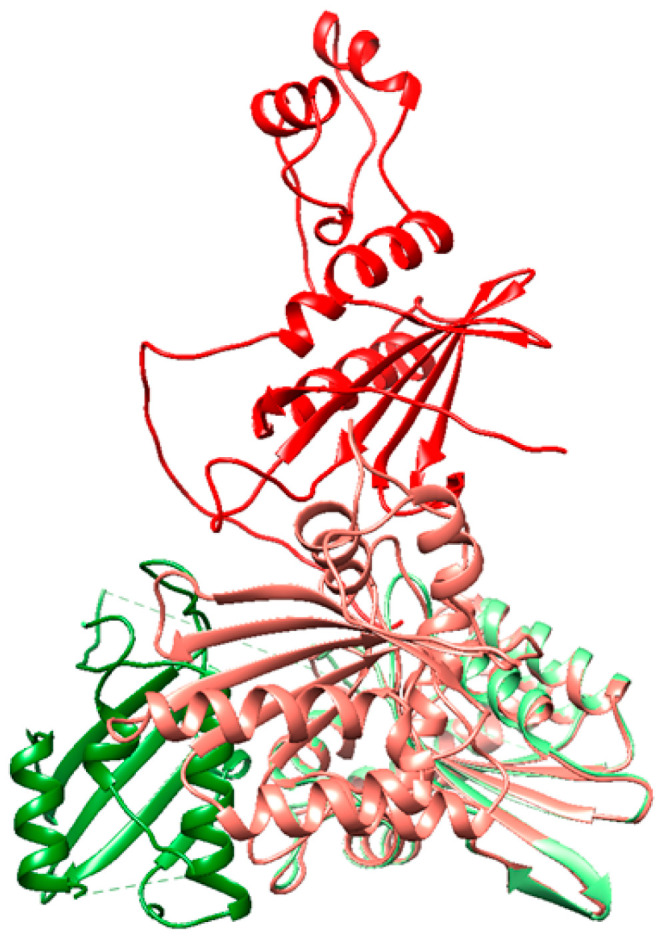
Spatial alignment of the structures of APOBEC3G complexes with Vif from PDB 8j62 (green) and those obtained using AlphaFold3 (red) (RMSD = 33.9 Å).

The most similar structures were the APOBEC3G complexes with Vif subtypes A1 and C (RMSD is 15.6 Å) and the UNG complexes with Vpr subtypes A1 and C (RMSD is 4.2 Å) (Figure S5). From the analysis of the number of contacts and binding energies, it follows that APOBEC3G binds to Vif A1 more strongly than to other Vif subtypes. Meanwhile, UNG binds to all the studied subtypes with approximately the same strength (Figure S6). Additional analysis showed that the structures of APOBEC3G complexes with Vif presented in the PDB and obtained using the AlphaFold3 program do not match (Figure 4). This can be explained by the fact that the structures placed in the PDB are missing a large number of aa residues: in the 8j62 (PDB ID) structure of APOBEC3G (chain A), aa residues 196 to 384 are missing, and in the structure of Vif (chain C), aa residues 1 to 2, 113 to 159, and 177 to 192 are missing.

**Figure 5:**
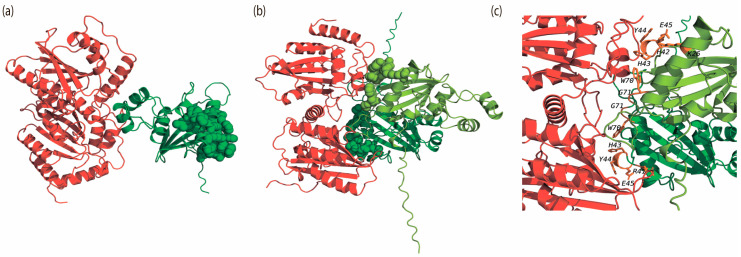
Tertiary complexes of APOBEC3G (red) with Vif B (green); (**a**) monomer, (**b**) dimer, (**c**) increased contact area, obtained using AlphaFold3. Amino acid residues (K^22^, K^26^, Y^30^, ^40^YRHHYE^45^, S^52^, W^70^, ^69^YXXL^72^, ^161^PPLP^164^) of the monomeric Vif B protein, which, according to the literature data [13,50], interact with APOBEC3G, are highlighted by spheres. Residues interacting with APOBEC3G in the Vif B dimer complex are shown as spheres (chain 1, light green: K^26^, ^42^HHYE^45^, ^70^WG^71^; chain 2, dark green: R^41^, ^43^HYE^45^, ^70^WG^71^) in panel (**b**) and as side chain residues (orange) in panel (**c**).

We also analyzed the complexes of APOBEC3G with the dimer, tetramer, and hexamer of Vif subtypes A1, B, C, and D. Table S5 shows the matrix of interactions between amino acid residues in the complexes of APOBEC3G and Vif, where 1 indicates an interaction of a residue with APOBEC3G and 0 indicates no interaction. The amino acid residues that, according to the literature, should interact with APOBEC3G are highlighted. The analysis of the contact matrix indicates that as the number of Vif monomers interacting with APOBEC3G increases, they begin to interact more with the N-terminal part than with the C-terminal part (Table S5). In the obtained models of APOBEC3G complexes with the Vif monomer, none of the amino acid residues of the Vif proteins that, according to the literature, should interact with APOBEC3G (K^22^, K^26^, Y^30^, ^40^YRHHYE^45^, S^52^, W^70^, ^69^YXXL^72^, ^161^PPLP^164^) were found to interact (Figure 5a). However, in the APOBEC3G complexes with Vif oligomers, such interactions were observed (see Figure 5b,c and Table S5): K^26^, ^42^HHYE^45^, ^70^WG^71^; R^41^, ^43^HYE^45^, ^70^WG^71^.

### 3.5. Wu–Kabat Protein Variability Index of the Vif and Vpr Proteins in the HIV-1 Group M

**Figure 6:**
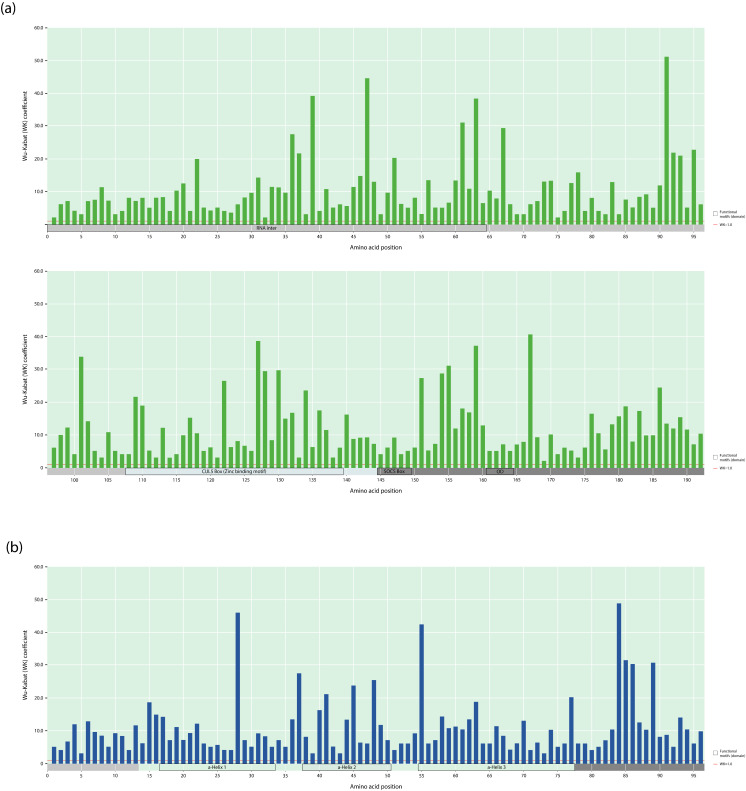
Amino acid residue variability landscape of the full-length Vif and Vpr proteins of HIV-1 group M. Rectangular bars represent the Wu–Kabat protein variability index for each amino acid residue in the Vif (**a**) and Vpr (**b**) proteins; the amino acid residues are numbered according to the reference strain HXB2, subtype B. The N-terminal regions of Vif and Vpr are marked in light gray, and the C-terminal regions—dark gray. Annotated protein motifs (domains) are shown as rectangles (above the scale): RNA inter, RNA interaction motif; CUL5 Box, Cullin 5 Box (HCCH zinc-binding motif); SOCS Box, suppressor of cytokine-signaling box; OD, oligomerization domain.

Among the 5,286 analyzed sequences, 100% of the Vif and Vpr protein positions had a Wu–Kabat (WK) index greater than 1 (Figure 6). The median WK coefficient for the full-length Vif (8.0) and Vpr (8.1) proteins were very close.

In our study, WK values of less than 10% were classified as low-level variability, from 10% to 20%—as intermediate-level variability, from 20% to 50%—as above-average variability, and 50% or more—as high-level variability. The maximum WK value for the Vif protein was 51.1, found at site 91 (K91Q/L/E/R/T/M), located in the N-terminal region, followed by site 47 (R47T/Q/P/N/I/H/S) in the RNA interaction motif (WK = 44.5) and site 167 (R47K/A/T/Q/S) in the C-terminal region (WK = 40.6). The highest WK coefficient was noted at site 91, which is involved in the nuclear transport inhibitory signal, while the highest intermediate value of the coefficient was observed at sites 39 and 63, which are involved in the interaction with APOBEC3H [12,13]. In regions associated with the interaction with APOBEC3G (K^26^, Y^30^, ^40^YRHHYE^45^, S^52^, W^70^, ^69^YXXL^72^, ^161^PPLP^164^), the coefficient values were approximately ±10, with one exception at position 22 (WK = 20). The lowest WK value was 2.0, recorded at sites 1 and 32 (RNA interaction), 75 (N-terminus), and 169 (C-terminus). The OD (4 amino acid residues) in the Vif protein had the lowest median WK value of 5.0. The SOCS Box (6 amino acid residues) exhibited slightly less variability than the RNA interaction motif (64 amino acid residues) and CUL5 Box (32 amino acid residues), with median WK values of 5.5, 7.1, and 8.2, respectively. Additionally, the N-terminal region (107 amino acid residues) was more conserved than the C-terminal region (49 amino acid residues), with median WK values of 7.2 versus 9.2.

## 4. Discussion

The genetic diversity of Vif and Vpr HIV-1 proteins is an interesting problem in the field of HIV research. In early studies, it has already been shown that the natural variability of Vif could influence its functional activity: it changes the effectiveness of its counteracting to cellular proteins of the APOBEC family [34]. In Mexican HIV-1 Vif sequences, genetic diversity was demonstrated in some functional motifs, including APOBEC binding sites [54]. In a molecular genetic analysis of HIV-1 variants circulating in Northern India, it was noted that Vif protein in circulating B/C recombinant forms had a higher ability to degrade APOBEC3G than Vif protein in the most prevalent HIV-1 C [31]. In a recent study, the genetic variations in HIV-1C Vif sequences from India and Uganda were compared. HIV-1C variants circulating in India are shown to have a higher predicted binding affinity to APOBEC3G than HIV-1C viruses in Uganda [42]. In a global study of Vif diversification over time, it was found that only 2% of sites changed over time, herewith patterns of diversity over time across HIV-1 clades showed slight differences [41]. The analysis of the viral diversification of HIV-1 in genes, and encoded accessory and regulatory viral proteins during primary HIV-1C infection demonstrated that in vif, there was a higher diversity observed than that in vpr [55]. In the molecular analysis of Vpr in virus variants circulated in South Africa, the highly conserved and variable motifs in a-helixes were distinguished, and the C-term was noted as more variable [43]. The comparison of HIV-1B Vpr genetic diversity between groups of patients with different clinical statuses in China has not resulted in significant differences detected in the functional domains [56]. The high conservation of Vpr was also noted in HIV-1 sub-subtype A6 variants [57,58]. At the same time, it was demonstrated that Vpr oligomeric forms of different genetic HIV-1 variants had some differences, and it was proposed that this may be associated with different functional properties [58]. This is the first globally Vif and Vpr genetic inter-clade diversity study in HIV-1 group M.

The comparison of amino acid residue variability for HIV-1 group M clades in Vif and Vpr revealed that sub-subtype A6 had the lowest amino acid diversity, while subtype B had the highest number of amino acid changes. This correlates with previous data on Rev protein diversity in HIV-1 group M clades and has been suggested to be related to different rates of dissemination of HIV-1 variants [59]. Additionally, it should be noticed that several researchers have suggested that differences in human HLA alleles could drive selection of different subtypes in different human populations [60]. Subtype B is spreading in different parts of the world: South and North America, North Africa and the Middle East, Europe, and Oceania, while sub-subtype A6 is distributed in Russia and the former Soviet Union [2,61]. However, human HLA distributions are not very different, and the evolution of the HIV-1 M group subtypes and sub-subtypes is mostly due to the epidemiological history of the spread of viruses around the world over time.

When analyzing changes in HIV-1 consensus sequences, the average degree of conservation in the HIV-1 group M was 86.4% for Vif and 91.3% for Vpr. It was previously found that for Rev, PR, RT, IN, and p24, this indicator was 80.8%, 93%, 94%, 96%, and 93.6%, respectively [59,62,63]. These results are consistent with the data on the rate of evolution: the lowest proportion of rate-shift sites was observed in Pol and Gag, the highest in Rev, and intermediate values in Vif and Vpr [60].

In Vif, the OD motif and SOCX box, as expected, were the most conserved regions [16,50], while the conservation of residues in the Vif domains (N-terminus, RNAinter, CUL5 Box, C-terminus) varied slightly between the HIV-1 clades (Figure 2b). The CUL5 Box, 108HX5CX17-18CX3-5H139, was the most variable region in Vif. Moreover, it was shown that mutations at positions located in this region can inhibit APOBEC3G degradation [64]. The N- and C-termins in Vif had an intermediate level of variability. The N-terminus contains regions involved in the regulation of Vif expression and in viral infectivity, 63RLVITTYW70, 86SIEW89, and 88EWRKKR93 [16]. In our study, R93 was one of the least conservative positions. The C-terminus is known to be disordered in nature and contains various functional motifs, including the last 25 residues involved in interaction with Pr55Gag, cytoplasmic membranes, and reverse transcriptase [16]. Moreover, in this region, the HIV-1 variants had different numbers of positions with conservation levels of 76–89%, 51–75%, and <50%. For example, in sub-subtype A6, only one position, 181, had a conservation level of 76–89%, while in subtype B, 7 positions (176, 178, 180, 184, 185, 189, and 190) had a conservation level of 76–89%, and one position, 186, had a conservation level of 51–75% (Figure 1). In Vpr, α-Helix-1 and the N-terminus had the lowest number of variable positions, which may be due to the involvement of these regions in the incorporation of Vpr into virions during the budding of viral particles from the surface of HIV-infected cells [65]. Some positions involved in the mutual orientation of three α-helices (26, 30, 38, 39, 42, 64) had a high level of conservation (Figure 1), while at positions 60 and 61, which were also involved in the mutual orientation of α-helices in seven and in three clades, respectively, the level of conservation was <75% (Figure 1). Moreover, at position 19, involved in the additional fixation of orientation of α-helices in 12 clades, the conservation level was 51–75% (Figure 1) [28]. Overall, the degree of conservation of the functional domains of Vif and Vpr varied across HIV-1 group M clades (Figure 2b,d), highlighting the importance of investigating the genetic diversity of these proteins in locally circulating virus variants.

In the HIV-1 consensus sequences, the substitutions were found at 23 positions in the Vif-interacting motifs (8, 17, 22, 30, 39, 41, 44, 45, 48, 52, 60, 61, 62, 63, 74, 77, 78, 90, 91, 92, 93, 96, 144) and at 5 positions in the Vpr-interacting motifs (61, 63, 66, 77, 85) (Figure 1). Moreover, some substitutions were associated with changes in the chemical properties of aa residues in the following cases: M8L/V, N22K/H, F39L/V/S, H48N, S52C, G60E/K/R, D61C/A/K, R63E/P/T, T74K, R90Q, K91L/Q, K92G/E, R93N/S/T/G, and S144P in Vif; Q66L, Q77R/H, and R85Q/P/I/L in Vpr [66,67]. Moreover, in the analysis of clade-specific substitutions in Vif, M8V and S52C were observed in 91_cpx (100%), F39S and R93T in 111_01C (100%), T74K in 63_02A6 (86–99%), R90Q in 91_cpx (70–85%), R93G in 137_0107 (70–85%), and R93S in 08_BC (70–85%); in Vpr, Q77R was observed in 42_BF1 (86–99%) (Figure 3). These data may be useful for the development of therapeutic drugs based on Vif and Vpr proteins.

In addition, several substitutions in the consensus sequences should be noted separately. Thus, the previously identified 22H in Vif was associated with low CD4+ cell counts and a higher viral load. In our study, it was observed in the consensus sequences of 11_cpx and 91_cpx (Figure 1) [44]. Q136P in Vif, and 41N and 55A in Vpr were previously associated with accelerated AIDS progression and more pronounced neurocognitive deficits, respectively [35,68]. Q136P was found in the consensus sequences of Vif in A6, 01_AE, 15_01B, 59_01B, 89_BF1, 103_01B, 111_01C, and 133_A6B clades; 41N in the consensus sequence of Vpr in clade 06_cpx; and 55A in the consensus sequences of Vpr in clade B and in recombinant forms 07_BC, 35_01D, 56_cpx, 66_BF1, 71_BF1, 85_BC, and 137_0107. Thus, natural amino acid substitutions in Vif and Vpr associated with HIV-infection progression may be subtype-specific.

Previous studies have shown differential anti-APOBEC3G activity of HIV-1 Vif proteins derived from different subtypes, with the Vif subtype C exhibiting the highest activity. It was also noted that HIV-1 subtype C is more protected from APOBEC3G [30]. In our study, bioinformatics analysis did not reveal a correlation between the subtypes and the spatial organization of the oligomeric structures of Vif and Vpr. Previous studies have suggested that the Vif multimerization process may be important for Vif functions: it may facilitate the formation of high-molecular ribonucleoprotein complexes and be involved in viral particle assembly, reverse transcription, APOBEC3G binding, and degradation [16,17,18]. In our study, bioinformatics analysis of APOBEC3G complexes with the dimer, tetramer, and hexamer of Vif subtypes A1, B, C, and D suggests that in nature, APOBEC3G may interact with Vif in oligomeric form.

Wu–Kabat analysis showed that a Wu–Kabat coefficient of variation (WK) of below 10, a low level, was present at 62% of sites in Vif and at 61.5% of sites in Vpr, whereas, as shown previously, in integrase (IN), reverse transcriptase (RT), and protease, 92%, 88%, and 44.5% of sites had WK below 10, respectively [62]. Thus, in Vif and in Vpr, group M viruses have an average level of evolutionary plasticity. In Vif, the positions with the highest WK values (39, 127, 151, and 167) correlate with sites under diversifying selection over time, confirming their high level of evolutionary plasticity [41]. At two sites, 39 and 63, involved in the interaction with APOBEC3H, the WK level was above average, and at position 91, it was involved in the nuclear transport inhibitory signal, and the WK level was also high. Thus, evolutionary plasticity at these sites may be associated with the regulation of Vif functional activity. Despite the fact that at position 52, involved in the interaction with APOBEC3G, WK had a low level of evolutionary plasticity, in the 91_cpx recombinant form at this position, the clade-specific substitution S52C was observed. Thus, substitutions at positions (39, 63, 91, and 52) may be of interest for further evaluation. In Vpr, there were no positions with a high level of WK. At the same time, all clade-specific amino acid residue substitutions were located at positions with intermediate and above-average levels of evolutionary plasticity.

The main limitation in this study is the low number of nearly full-genome sequences for some HIV-1 variants in the Los Alamos National Laboratory HIV database, but the inclusion of partial sequences in the analysis may lead to a high risk of bias.

## 5. Conclusions

For the first time, a comprehensive analysis of Vif and Vpr genetic diversity in HIV-1 group M clades was carried out. The average level of conservation within HIV-1 group M was 86.4% for Vif and 91.3% for Vpr. In both proteins, sub-subtype A6 showed the lowest amino acid diversity, while subtype B had the highest number of amino acid residue changes. In general, the level of conservation in the functional domains in Vif and Vpr varied across clades. Substitutions in consensus sequences, as well as clade-specific substitutions located in interacting motifs and associated with the changes in the chemical properties of residues, were noted. Moreover, amino acid substitutions in consensus sequences previously associated with disease progression were found in some subtypes. There was no correlation between the subtypes and the spatial organization of the oligomeric structures. Bioinformatics analysis confirmed that Vif can interact with APOBEC3G in oligomeric form. The obtained data may be useful for the development of new therapeutic agents and for understanding the influence of clade-specific features on pathogenesis.

## Figures and Tables

**Table 1:** Natural variations in the full-length Vif|Vpr proteins within individual HIV-1 group M clades.

Clade ^a^	Number of Near Full-Length HIV-1 Genomes in the HIV Database	Number of Downloaded HIV-1 Sequences Used in Consensus	Number of Changes (Vif|Vpr)			Mean Changes per Sequence (Vif|Vpr) ^c^	Variable Positions (Vif|Vpr) (%)
			Insertions ^b^	Deletions	Substitutions		
A1	813	250	2|49	13|21	4595|1755	18.4|7.1	75.0|72.9
A6	235	221	0|6	3|21	2753|1075	12.5|5.0	67.7|60.4
B	11,407	1888	86|135	137|306	36,917|13,881	19.6|7.5	94.3|97.9
C	2449	878	19|10	29|51	12,377|6083	14.1|7.0	92.2|97.9
D	225	160	4|0	3|5	1855|1072	11.6|6.7	62.0|70.8
F1	79	73	7|2	2|4	1086|404	14.9|5.6	52.6|46.8
F2	14	14	1|0	0|0	163|93	11.6|6.6	30.0|26.0
G	101	89	5|13	140|7	1603|652	19.6|7.4	64.6|58.3
H	10	10	1|0	0|1	180|65	18.0|6.6	36.5|28.1
01_AE	2275	613	8|43	132|106	8373|3206	13.9|5.4	84.4|84.4
02_AG	233	205	8|0	17|5	3725|1198	18.3|5.9	76.0|69.8
06_cpx	19	17	0|0	2|0	243|112	14.4|6.6	37.0|40.6
07_BC	48	43	0|1	5|1	390|200	9.2|4.7	39.4|41.7
08_BC	37	33	0|0 (*)	7|1	297|111	9.2|3.4	48.2|44.8
11_cpx	25	24	3|0	2|3	357|107	15.0|4.6	43.2|30.2
12_BF	14	14	1|0	0|0	158|65	11.3|4.6	32.8|32.3
13_cpx	10	10	5|0	1|2	124|47	12.5|4.9	27.6|22.9
14_BG	14	12	0|0	0|0	93|37	7.8|3.1	20.8|15.6
15_01B	8	8	0|7	0|1	87|43	10.9|5.5	22.9|27.1
22_01A1	21	15	0|0	1|1	228|77	15.3|5.2	35.9|30.2
35_A1D	22	22	0|0	0|0	198|79	9.0|3.6	38.0|28.1
42_BF1	17	15	0|0	0|0	53|80	3.5|5.3	9.4|10.4
46_BF1	8	8	0|0	0|0	76|36	9.5|4.5	23.4|21.9
56_cpx	8	8	0|0	0|0	91|40	11.4|5.0	27.1|19.8
59_01B	9	8	0|0	0|0	38|9	4.8|1.1	9.9|5.2
63_02A6	23	22	0|15	0|0	177|56	8.0|2.5	27.1|22.9
64_BC	9	8	0|0 (*)	0|0	48|21	6.0|2.6	14.0|16.7
66_BF1	8	8	0|2	0|2	102|52	12.8|6.8	24.5|25.0
71_BF1	14	13	0|0	0|0	218|87	16.8|6.7	38.0|34.4
85_BC	11	11	0|0 (*)	1|0	71|36	6.5|3.3	20.7|21.9
89_BF1	9	9	2|0	0|0	95|42	10.6|4.7	23.4|21.9
91_cpx	10	10	0|0	0|2	55|10	5.5|1.2	13.5|6.25
103_01B	10	10	0|0	0|0	85|29	8.5|2.9	19.8|13.5
111_01C	8	8	0|0	0|0	58|35	7.3|4.4	19.3|19.8
133_A6B	8	8	0|0	0|1	48|14	6.0|1.9	16.7|9.4
137_0107	9	9	0|0 (*)	0|0	53|21	5.9|2.3	17.1|14.6
145_0755	10	10	0|0 (*)	0|0	28|27	2.8|2.7	7.8|14.6

^a^—Only HIV-1 subtypes or CRFs with more than 8 genomic sequences are listed. ^b^—HIV-1 subtypes or CRFs with insertions of one aa residue (Asparagine, N) between positions 141 and 142 of the Vif protein are marked with an asterisk. ^c^—Includes aa residue changes and deletions.

## Data Availability

The data presented in this study are available on request from the corresponding author.

## Supplementary Materials

The following supporting information can be downloaded at https://www.mdpi.com/article/10.3390/v18010116/s1, Figure S1. Average amino acid residue diversity in the full-length Vif and Vpr protein within individual HIV-1 group M clades; Figure S2. Spatial alignment of the structures of Vif dimers: for subtypes; Figure S3. Spatial alignment of the structures of Vif tetramers: for subtypes; Figure S4. Spatial alignment of the structures of Vif hexamers: for subtypes; Figure S5. Spatial alignment of the structures of APOBEC3G complexes with Vif subtypes A1 and C (A) and UNG complexes with Vpr subtypes A1 and C (B); Figure S6. The number of contacts (contact distance 5Å) and binding energy (taken with a positive sign) between structures in the complexes of APOBEC3G with Vif (A) and UNG with Vpr (B); Table S1. GenBank Accession numbers, origin information and sampling date for the HIV-1 sequences used in this study; Table S2. The number of nearly full-genome HIV sequences from the Los Alamos HIV database for a Vif/Vpr protein analyzed in the present study; Table S3. Natural variations at the individual domain of the Vif and Vpr protein within difference HIV-1 group M clades; Table S4. Range of conservation of individual Vif and Vpr motif/domain across different HIV-1 group M clades; Table S5. Matrix of contacts of APOBEC3G with monomer, dimer, tetramer and hexamer of vifs A1, B, C, and D.
