# Supplementary material for: Genetic Diversity of Vif and Vpr Accessory Proteins in HIV-1 Group M Clades
Source: Viruses. 2026 Jan 15;18(1):116. doi: 10.3390/v18010116 (PMC12846688; doi:10.3390/v18010116)
Supplement: Supplementary file 1 [file viruses-18-00116-s001.zip › Figures_supple.pdf]

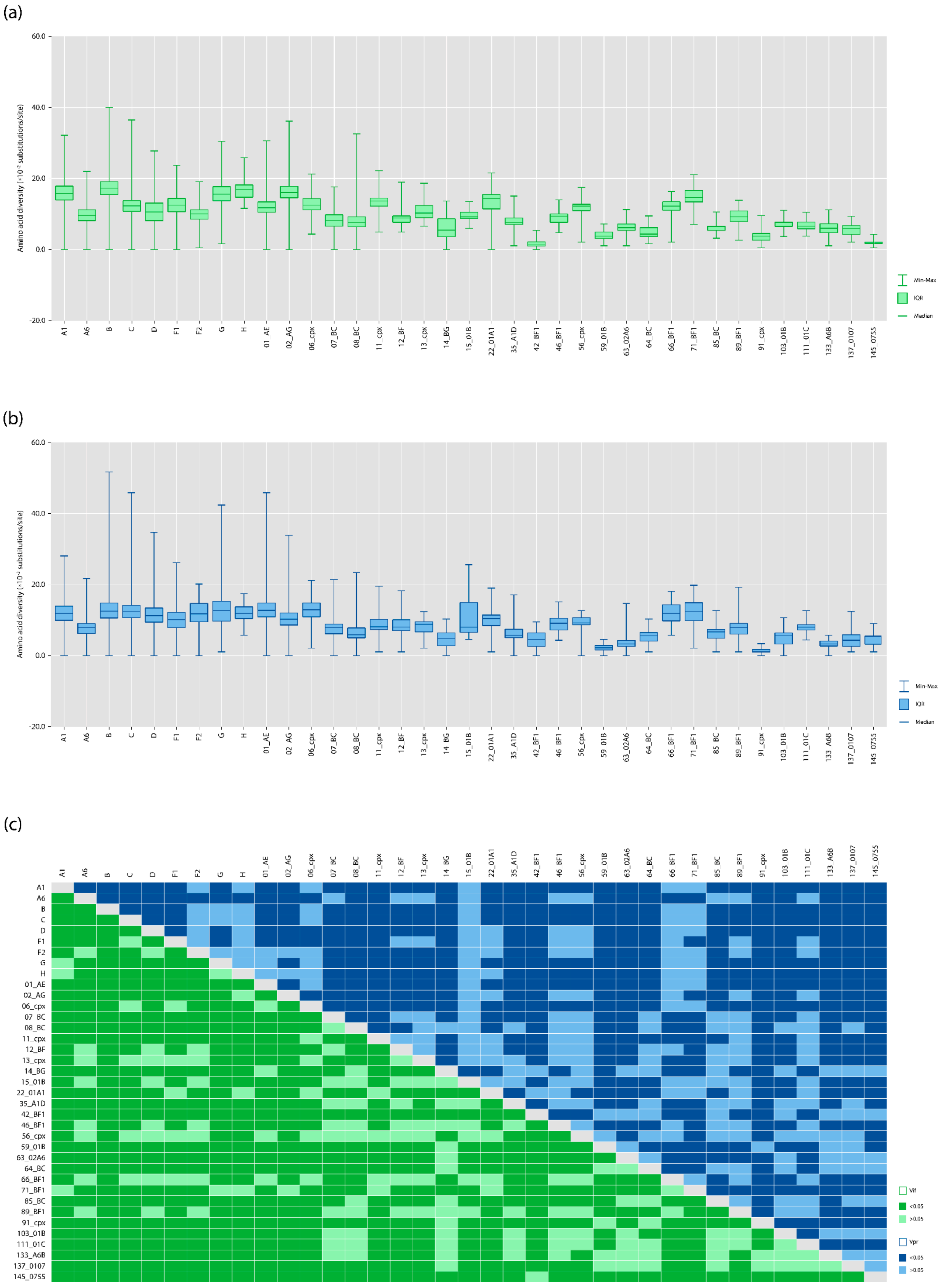

Figure S1. Average amino acid residue diversity in the full-length Vif and Vpr protein within individual HIV-1 group M clades. Box plots (**a** and **b**) show the distribution of amino acid residue diversity between individual HIV-1

sequences within different HIV-1 clades. Matrix of p-values (**c**) for the pairwise comparisons of the amino acid diversity in different HIV-1 clades was estimated using the Mann–Whitney U-test with Bonferroni multiple-test correction ($p=\alpha/m$, with $\alpha= 0.05$, m=1332 tests; the p-value is less or larger than a threshold (0.05) are displayed using a color gradient, as described in the legend. Min, minimum; Max, maximum; IQR, interquartile range.

(a)

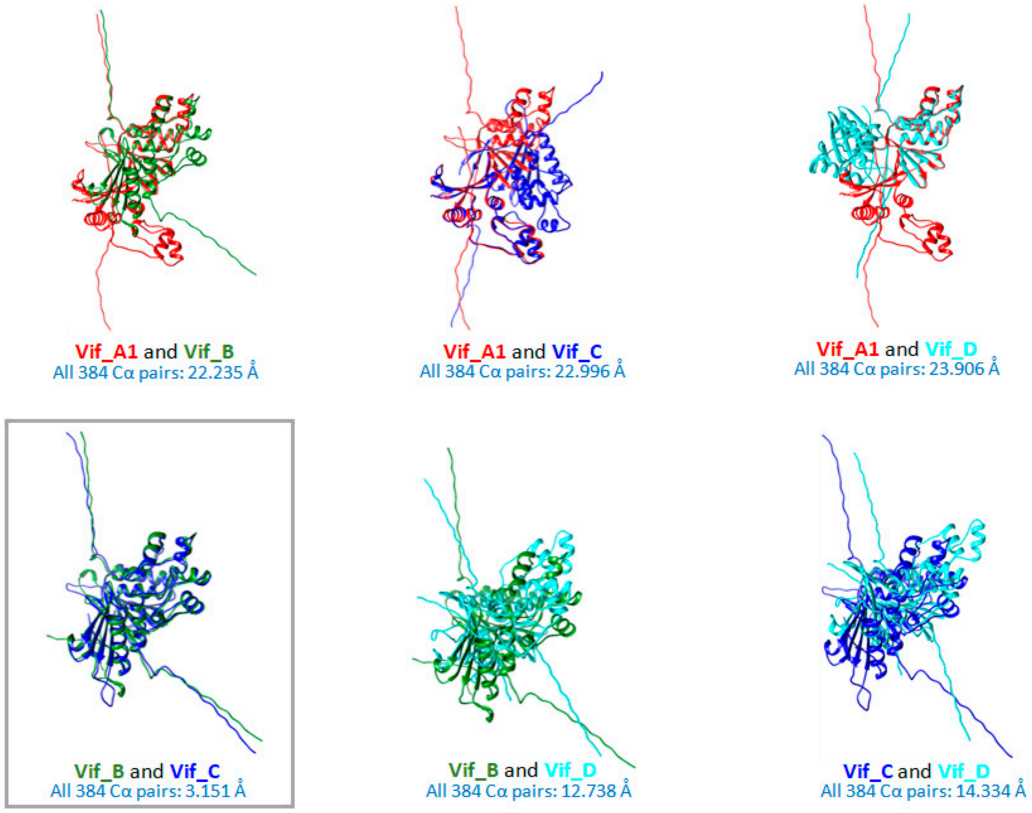

(b)

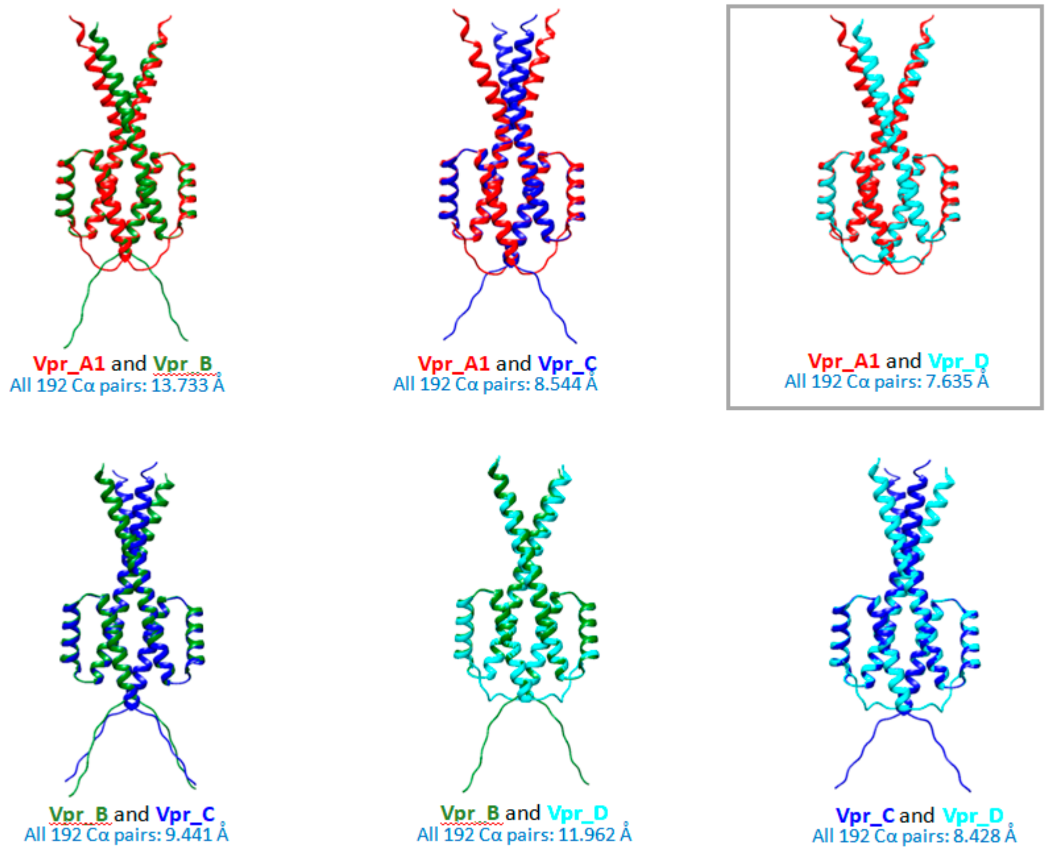

Figure S2. Spatial alignment of the structures of Vif dimers: for subtypes A1, B, C and D (a)**.** Spatial alignment of the structures of Vpr dimers: for subtypes A1, B, C and D (b).

(a)

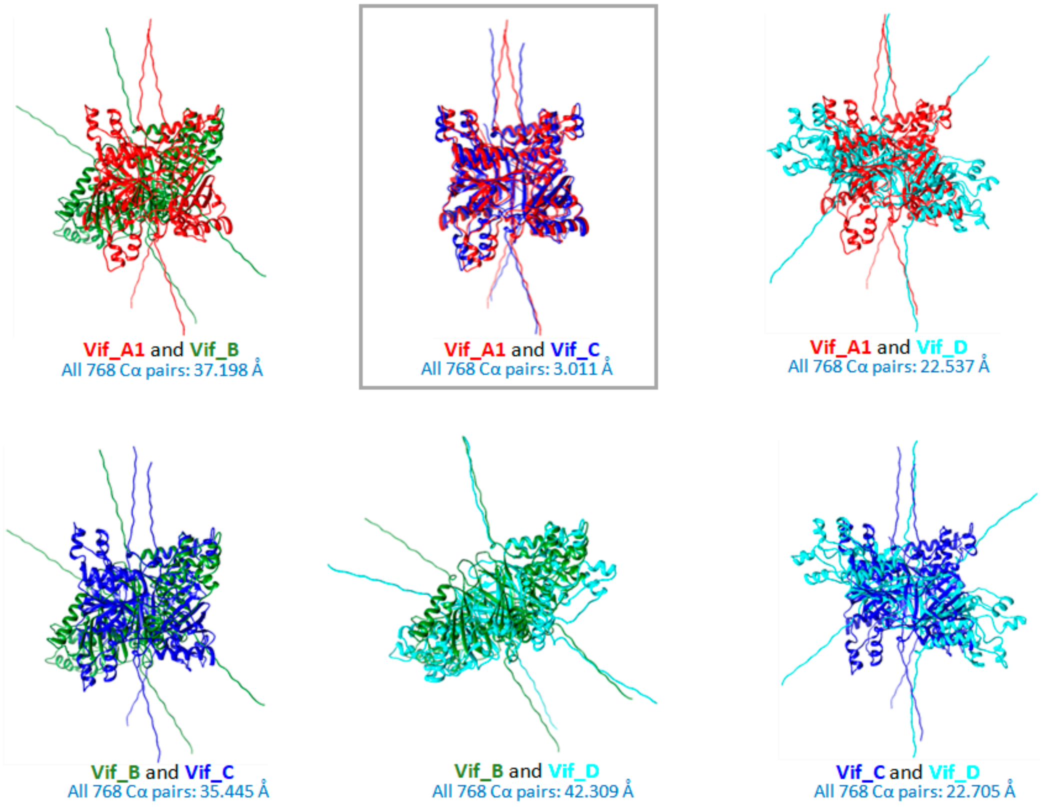

(b)

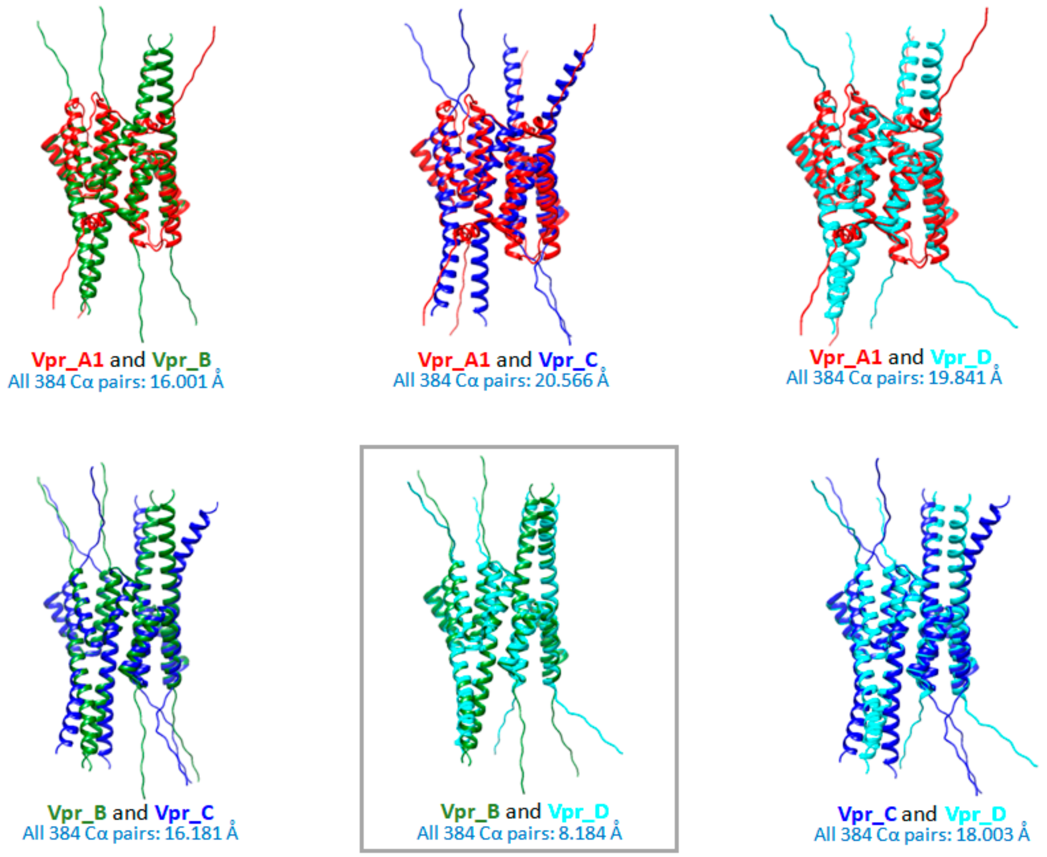

Figure S3. Spatial alignment of the structures of Vif tetramers: for subtypes A1, B, C and D (a)**.** Spatial alignment of the structures of Vpr tetramers: for subtypes A1, B, C and D (b).

(a)

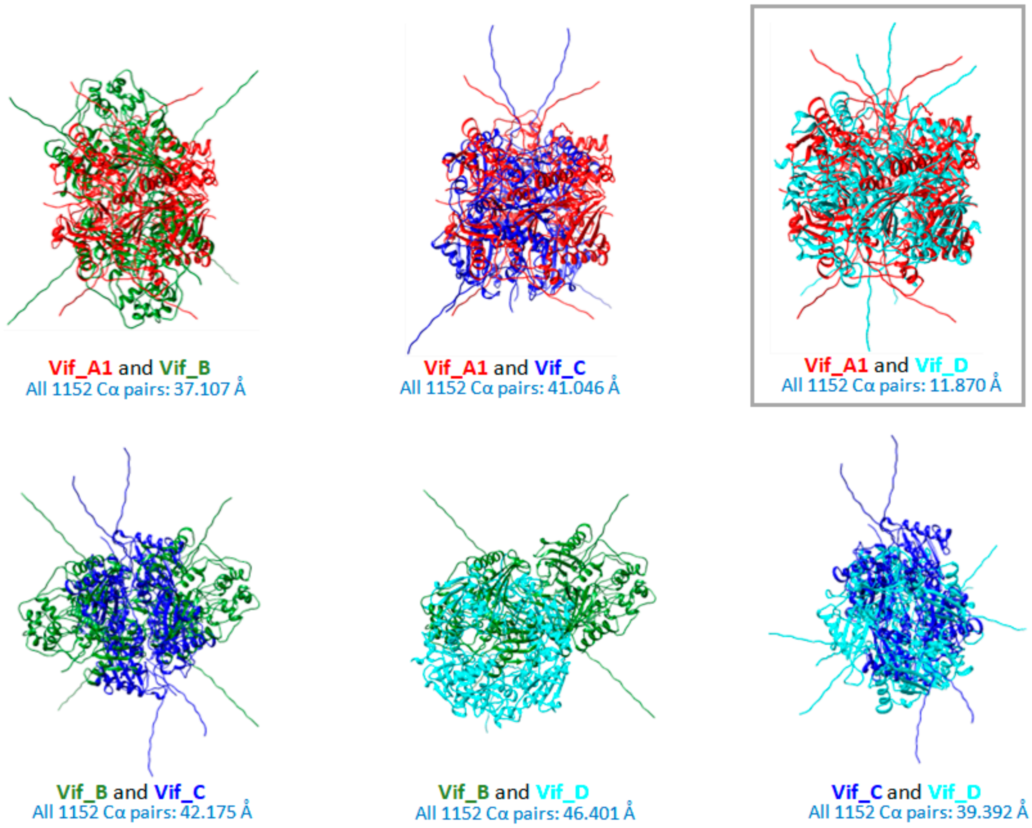

(b)

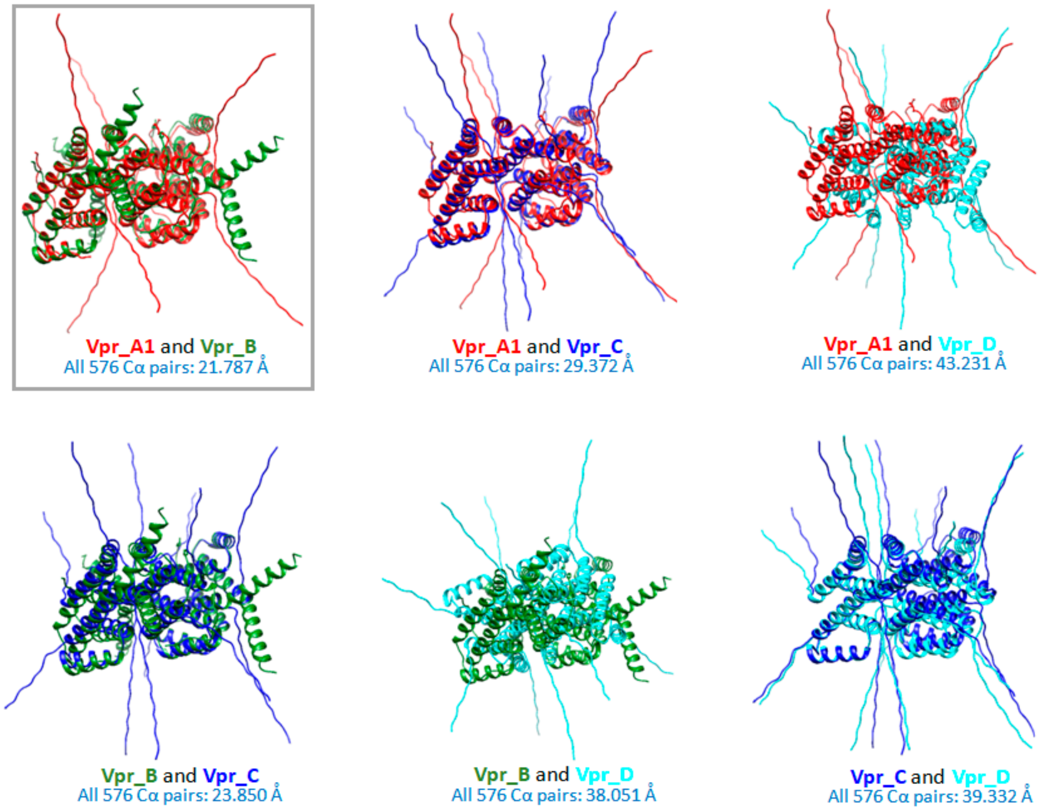

Figure S4. Spatial alignment of the structures of Vif hexamers: for subtypes A1, B, C and D (a)**.** Spatial alignment of the structures of Vpr hexamers: for subtypes A1, B, C and D (b).

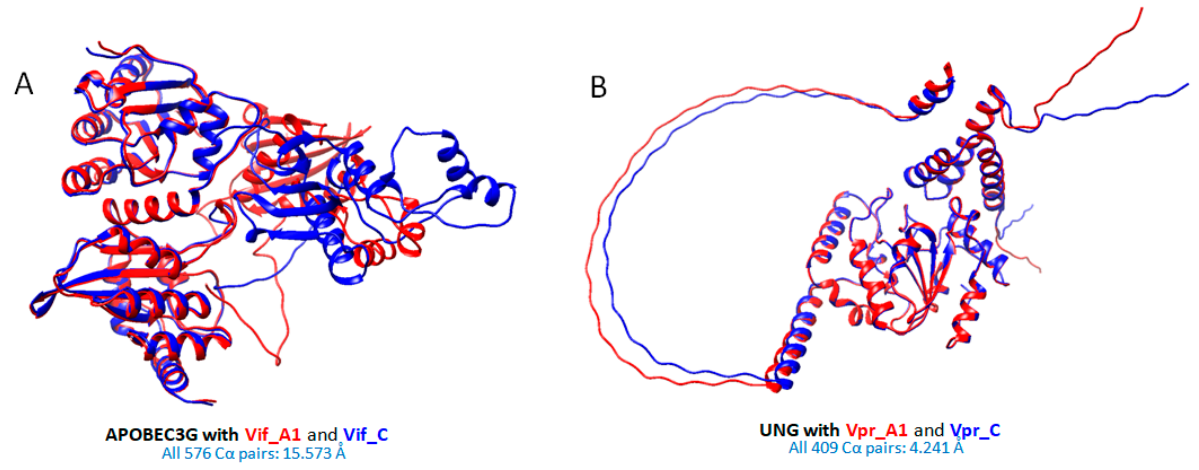

Figure S5. Spatial alignment of the structures of APOBEC3G complexes with Vif subtypes A1 and C (A) and UNG complexes with Vpr subtypes A1 and C (B).

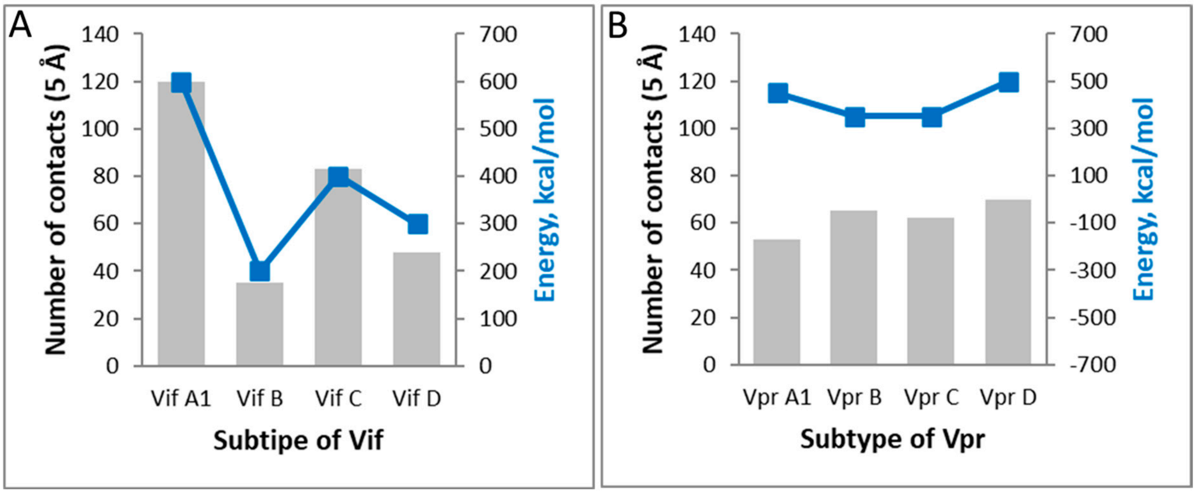

Figure S6. The number of contacts (contact distance 5Å) and binding energy (taken with a positive sign) between structures in the complexes of APOBEC3G with Vif (A) and UNG with Vpr (B).
