# Supplementary material for: Genetic Diversity of Vif and Vpr Accessory Proteins in HIV-1 Group M Clades
Source: Viruses. 2026 Jan 15;18(1):116. doi: 10.3390/v18010116 (PMC12846688; doi:10.3390/v18010116)
Supplement: Supplementary file 1 [file viruses-18-00116-s001.zip › Table_S2.pdf]

Supplementary Table S2. The number of nearly full-genome HIV sequence from the Los Alamos HIV database for a Vif/Vpr protein analyzed in the present study (data available as of December 28, 2024).

HIV-1 form		No. sequences [a]	HIV-1 form	No. sequences	HIV-1 form	No. sequences
Sub(-sub)types	A1	813/250	46_BF1	8/8	111_01C	8/8
	A2	4/4	47_BF	3/3	112_0107	4/4
	A3	3/3	48_01B	3/3	113_01B	3/3
	A4	3/3	49_cpx	4/4	114_01B	7/7
	A6	235/221	50_A1D	5/5	115_01C	2/2
	A7	3/3	51_01B	7/7	117_0107	3/3
	A8	2/2	52_01B	3/3	118_BC	1/1
	B	11407/1888	53_01B	4/4	119_0107	7/7
	C	2449/878	54_01B	3/3	120_0107	3/3
	D	225/160	55_01B	7/7	121_0107	4/4
	F1	79/73	56_cpx	8/8	122_BF1	5/4
	F2	14/14	57_BC	9/7	123_0107	3/3
	G	101/89	58_01B	6/6	124_cpx	2/2
	H	10/10	59_01B	9/8	125_0107	4/4
	J	7/7	60_BC	5/5	126_0755	4/4
	K	2/2	61_BC	4/3	127_07109	3/3
	L	3/3	62_BC	3/3	128_07B	4/4
CRFs	01_AE	2275/613	63_02A	30/22	129_56G	7/7
	02_AG	233/205	64_BC	9/8	130_A1B	7/2
	03_AB	4/4	65_cpx	7/7	131__A1B	1/1
	04_cpx	5/5	66_BF1	8/8	132_94B	5/5
	05_DF	4/4	67_01B	2/2	133_A6B	8/8
	06_cpx	19/17	68_01B	5/5	134_0107	6/6
	07_BC	48/43	69_01B	7/7	135_0107	6/5
	08_BC	37/33	70_BF1	4/3	136_0107	3/3
	09_cpx	5/5	71_BF1	14/13	137_0107	9/9
	10_CD	3/3	72_BF1	7/6	138_cpx	3/3
	11_cpx	25/24	73_BG	2/2	139_02B	4/4
	12_BF	14/14	74_01B	3/3	140_0107	18/5
	13_cpx	10/10	75_BF1	5/5	141_BF1	4/4
	14_BG	14/12	76_01B	3/3	142_BC	5/5
	15_01B	8/8	77_cpx	4/4	143_cpx	3/3
	16_A2D	4/4	79_0107	4/4	145_0755	10/10
	17_BF	7/7	80_0107	3/3	146_BC	3/3
	18_cpx	7/7	81_cpx	2/2	147_A6B	3/3
	19_cpx	5/5	84_A1D	3/3	149_01B	2/2
	20_BG	5/5	85_BC	11/11	150_07149	4/4
	21_A2D	3/3	87_cpx	3/3	151_0107	3/3
	22_01A1	21/15	88_BC	3/3	152_DG	5/5
	23_BG	2/2	89_BF1	9/9	153_55B	4/4
	24_BG	4/4	90_BF1	6/6	154_0755	3/3
	25_cpx	6/6	91_cpx	10/10	155_0755	3/3
	26_A5U	4/4	92_C2U	7/7	156_0755	5/5
	27_cpx	4/4	93_cpx	3/3	157_A6C	3/3

28_BF	5/5	94_cpx	6/6	158_0107	4/4
29_BF	6/6	95_02B	3/3	159_01103	3/3
31_BC	3/3	96_cpx	3/3		
32_06A6	3/3	97_01B	5/5		
33_01B	7/7	98_06B	1/1		
34_01B	3/3	99_BF	2/2		
35_AD	22/22	100_01C	3/3		
36_cpx	3/3	101_01B	3/3		
37_cpx	4/4	102_0107	2/2		
38_BF	5/5	103_01B	10/10		
39_BF	3/3	104_0107	3/3		
40_BF	4/4	105_0108	5/5		
41_CD	3/3	106_cpx	6/6		
42_BF	17/17	107_01B	4/4		
43_02G	5/5	108_BC	5/5		
44_BF	3/3	109_0107	5/5		
45_cpx	5/5	110_BC	3/3		

[a] Number nearly full-length HIV-1 genomes/number sequences used in consensus calculation (See «Material and Methods»). HIV-1 subtypes/CRFs with more than available 8 sequences marked in red. CRF, circulating recombinant forms.
