# Supplementary material for: Genetic Diversity of Vif and Vpr Accessory Proteins in HIV-1 Group M Clades
Source: Viruses. 2026 Jan 15;18(1):116. doi: 10.3390/v18010116 (PMC12846688; doi:10.3390/v18010116)
Supplement: Supplementary file 1 [file viruses-18-00116-s001.zip › Table_S3.pdf]

Supplementary Table S3. Natural variations at the individual domain of the Vif and Vpr protein within difference HIV-1 group M clades.

HIV-1 clade [a]	Vif protein - Mean changes per sequence (variable positions [b], %)					
	N-term (107 aa)	RNA inter (68 aa)	CUL5 Box (32 aa)	SOCS Box (6 aa)	OD (4 aa)	C-term (49 aa)
A1	8.7 (74.8)	5.4 (78.1)	3.6 (75.0)	<0.1 (16.7)	0 (0)	5.9 (75.5)
A6	7.4 (62.6)	4.8 (65.6)	2.7 (17.9)	0.1 (33.3)	<0.1 (25.0)	2.0 (75.5)
B	10.3 (96.3)	6.7 (96.9)	3.8 (90.6)	<0.1 (100.0)	<0.1 (75.0)	5.2 (91.8)
C	8.3 (90.6)	5.4 (93.7)	2.2 (90.6)	<0.1 (100.0)	<0.1 (100.0)	3.5 (95.9)
D	6.3 (61.7)	5.0 (65.6)	2.6 (59.3)	0 (0)	0 (0)	2.5 (65.3)
F1	8.2 (48.6)	5.9 (54.7)	3.2 (56.2)	<0.1 (16.7)	0 (0)	3.3 (57.1)
F2	6.5 (28.0)	4.5 (28.1)	2.1 (37.5)	0 (0)	<0.1 (25.0)	2.8 (26.5)
G	8.5 (60.7)	5.2 (60.9)	3.0 (65.6)	0 (0)	<0.1 (25.0)	6.2 (69.4)
H	9.5 (32.7)	7.0 (37.5)	3.5 (40.6)	<0.1 (16.7)	0 (0)	4.3 (40.8)
01_AE	7.7 (75.7)	4.1 (81.2)	2.5 (87.5)	<0.1 (83.3)	<0.1 (50.0)	3.5 (85.7)
02_AG	9.1 (75.7)	5.4 (78.1)	3.3 (71.9)	<0.1 (50.0)	<0.1 (50.0)	5.6 (79.6)
06_cpx	7.5 (28.0)	4.9 (31.2)	2.8 (50.0)	0 (0)	0 (0)	4.0 (46.9)
07_BC	5.6 (35.5)	3.6 (32.8)	0.6 (31.2)	0 (0)	0 (0)	2.5 (53.1)
08_BC	5.4 (51.4)	3.9 (54.7)	1.0 (37.5)	<0.1 (33.3)	0 (0)	2.3 (46.9)
11_cpx	7.0 (37.4)	5.0 (39.1)	2.6 (46.9)	0 (0)	0 (0)	5.0 (53.1)
12_BF	5.8 (26.2)	4.4 (28.1)	2.1 (37.5)	0 (0)	0 (0)	3.3 (46.9)
13_cpx	7.4 23.4)	5.2 (25.0)	1.7 (31.2)	0 (0)	0 (0)	3.0 (32.6)
14_BG	3.7 (17.7)	2.1 (18.7)	1.8 (28.1)	0 (0)	0 (0)	1.7 (20.4)
15_01B	6.0 (22.4)	3.7 (25.0)	2.4 (21.9)	0 (0)	0 (0)	2.5 (26.5)
22_01A1	8.4 (33.6)	4.9 (32.8)	3.2 (40.6)	0 (0)	0 (0)	3.2 (38.8)
35_A1D	5.7 (34.6)	3.5 (35.9)	1.5 (53.1)	0 (0)	0 (0)	1.6 (34.7)
42_BF1	3.0 (14.0)	1.5 (15.6)	0 (0)	0 (0)	0 (0)	0.3 (6.1)

46_BF1	4.6 (21.5)	3.5 (25.0)	2.4 (31.2)	0 (0)	0 (0)	2.1 (20.4)
56_cpx	5.5 (26.2)	3.5 (26.6)	2.1 (25.0)	0 (0)	0 (0)	3.6 30.6)
59_01B	2.9 (10.3)	2.1 (12.5)	0.4 (6.2)	0 (0)	0 (0)	1.5 (12.2)
63_02A6	3.7 (22.4)	2.4 (20.3)	3.0 (50.0)	0.1 (16.7)	0 (0)	0.9 (18.4)
64_BC	4.1 (14.9)	1.6 (10.9)	0.2 (6.2)	0 (0)	0 (0)	1.6 (16.3)
66_BF1	7.1 (24.3)	3.9 (23.4)	1.6 (21.9)	0.1 (16.7)	0 (0)	4.0 (28.6)
71_BF1	8.5 (32.7)	5.6 (31.2)	3.3 (46.9)	0.1 (16.7)	0 (0)	4.8 (44.9)
85_BC	3.3 (17.7)	1.5 (14.1)	1.0 (18.7)	0 (0)	0 (0)	1.9 (26.5)
89_BF1	6.3 (19.6)	4.0 (18.7)	1.9 (34.4)	0.1 (16.7)	0 (0)	2.1 (24.5)
91_cpx	2.9 (11.2)	2.4 (17.2)	1.2 (18.7)	0 (0)	0 (0)	1.4 (16.3)
103_01B	5.2 (20.6)	2.6 (15.6)	1.1 (18.7)	0.2 (16.7)	0 (0)	2.1 (18.4)
111_01C	4.5 (22.4)	3.0 (23.4)	0.4 (6.2)	0 (0)	0 (0)	2.4 (22.4)
133_A6B	3.2 (16.8)	2.4 (20.3)	1.0 (18.7)	0.4 (16.7)	0 (0)	1.5 (14.3)
137_0107	3.9 (18.7)	2.9 (21.9)	0.4 (25.0)	0 (0)	0 (0)	1.5 (20.4)
145_0755	1.1 (5.6)	0.7 (4.7)	0.3 (28.1)	0 (0)	0 (0)	1.4 (12.2)

HIV-1 clade [a]	Vpr protein - Mean changes per sequence (variable positions [b], %)				
	N-term (13 aa)	aHelix-1 (17 aa)	aHelix-2 (13 aa)	aHelix-30.2 (23)	C-term (19 aa)
A1	0.3 (0.0)	1.4 (0.0)	0.8 (0.0)	1.3 (0.0)	2.8 (0.0)
A6	0.1 (0.0)	0.9 (0.0)	0.7 (0.0)	1.0 (0.0)	1.5 (0.0)
B	0.4 (0.0)	1.1 (0.0)	1.5 (0.0)	1.9 (0.0)	2.1 (0.0)
C	0.7 (0.0)	0.8 (0.0)	1.4 (0.0)	2.2 (0.0)	1.5 (0.0)
D	0.3 (0.0)	0.7 (0.0)	1.1 (0.0)	1.4 (0.0)	2.3 (0.0)
F1	0.5 (0.0)	1.1 (0.0)	0.9 (0.0)	1.7 (0.0)	1.3 (0.0)
F2	0 (0.0)	0.4 (0.0)	1.2 (0.0)	2.0 (0.0)	2.2 (0.0)
G	0.7 (0.0)	0.9 (0.0)	1.1 (0.0)	1.1 (0.0)	3.1 (0.0)
H	0.3 (0.0)	0.8 (0.0)	0.9 (0.0)	1.9 (0.0)	1.6 (0.0)
01_AE	0.9 (0.0)	0.7 (0.0)	1.0 (0.0)	1.0 (0.0)	1.0 (0.0)
02_AG	0.6 (0.0)	0.8 (0.0)	0.9 (0.0)	1.6 (0.0)	1.6 (0.0)
06_cpx	0.3 (0.0)	1.1 (0.0)	1.7 (0.0)	1.8 (0.0)	1.2 (0.0)
07_BC	0.5 (0.0)	0.2 (0.0)	1.1 (0.0)	1.4 (0.0)	0.7 (0.0)
08_BC	0.3 (0.0)	0.3 (0.0)	0.3 (0.0)	1.3 (0.0)	0.7 (0.0)
11_cpx	0.1 (0.0)	0.4 (0.0)	0.8 (0.0)	0.8 (0.0)	1.6 (0.0)
12_BF	0.8 (0.0)	0.6 (0.0)	0.8 (0.0)	1.0 (0.0)	1.1 (0.0)
13_cpx	0.1 (0.0)	0.7 (0.0)	1.1 (0.0)	2.0 (0.0)	0.8 (0.0)
14_BG	0.1 (0.0)	0.7 (0.0)	0.7 (0.0)	0.7 (0.0)	0.8 (0.0)
15_01B	1.0 (0.0)	0.9 (0.0)	1.2 (0.0)	0.9 (0.0)	1.1 (0.0)
22_01A1	0.3 (0.0)	0.3 (0.0)	1.2 (0.0)	0.7 (0.0)	1.5 (0.0)
35_A1D	0.3 (0.0)	0.4 (0.0)	0.5 (0.0)	1.6 (0.0)	0.7 (0.0)
42_BF1	0 (0.0)	0 (0.0)	0.3 (0.0)	2.5 (0.0)	2.4 (0.0)
46_BF1	0.5 (0.0)	0.6 (0.0)	1.1 (0.0)	0.6 (0.0)	1.4 (0.0)

56_cpx	0.1 (0.0)	0.2 (0.0)	0.5 (0.0)	1.0 (0.0)	2.4 (0.0)
59_01B	0.2 (0.0)	0.0 (0.0)	0.6 (0.0)	0.2 (0.0)	0.0 (0.0)
63_02A6	<0.1 (0.0)	0.4 (0.0)	0.7 (0.0)	0.7 (0.0)	0.7 (0.0)
64_BC	0.6 (0.0)	0.4 (0.0)	0.2 (0.0)	0.6 (0.0)	0.7 (0.0)
66_BF1	0.5 (0.0)	0.9 (0.0)	0.6 (0.0)	1.4 (0.0)	2.0 (0.0)
71_BF1	0.2 (0.0)	1.2 (0.0)	1.2 (0.0)	1.5 (0.0)	2.1 (0.0)
85_BC	0.1 (0.0)	0.3 (0.0)	0.7 (0.0)	1.0 (0.0)	0.8 (0.0)
89_BF1	0.4 (0.0)	0.3 (0.0)	0.3 (0.0)	1.3 (0.0)	1.8 (0.0)
91_cpx	0 (0.0)	0.1 (0.0)	0.2 (0.0)	0.1 (0.0)	0.7 (0.0)
103_01B	0.1 (0.0)	0.4 (0.0)	0.6 (0.0)	0.9 (0.0)	0.4 (0.0)
111_01C	0.2 (0.0)	0.6 (0.0)	1.5 (0.0)	0.4 (0.0)	1.1 (0.0)
133_A6B	0 (0.0)	0 (0.0)	0.5 (0.0)	0.6 (0.0)	0.4 (0.0)
137_0107	0.5 (0.0)	0.5 (0.0)	0 (0.0)	1.0 (0.0)	0 (0.0)
145_0755	0.2 (0.0)	0.4 (0.0)	0.6 (0.0)	0.9 (0.0)	0.3 (0.0)

[a] - Only HIV-1 subtypes or CRFs with more than 8 genomic sequences are listed.

[b] - Includes aa changes and deletions.

Annotated protein motif/domain: N-term, N-terminal region; C-term, C-terminal region; RNA inter, RNA interaction motif, CUL 5 Box, Cullin 5 box (HCCH zinc-binding motif); SOCS Box, suppressor of cytokine signaling box; OD, oligomerization domain.
