# Supplementary material for: Genetic Diversity of Vif and Vpr Accessory Proteins in HIV-1 Group M Clades
Source: Viruses. 2026 Jan 15;18(1):116. doi: 10.3390/v18010116 (PMC12846688; doi:10.3390/v18010116)
Supplement: Supplementary file 1 [file viruses-18-00116-s001.zip › Table_S4.pdf]

Table S4. Range of conservation of individual Vif and Vpr motif/domain across different HIV-1 group M clades.

Clade	Vif protein - Conservation (%)							
	82.0-83.9	84.0-85.9	86.0-87.9	88.0-89.9	90.0-91.9	92.0-93.9	94.0-95.9	96.0-97.9	98.0-100
Group M	CUL5 Box	C-term		N-term, RNA inter					SOCS Box, OD
A1			C-term	CUL5 Box	N-term, RNA inter				SOCS Box, OD
A6					CUL5 Box	N-term, RNA inter	C-term	SOCS Box	
B				RNA inter, CUL5 Box, C-term	N-term				SOCS Box, OD
C					RNA inter	N-term, CUL5 Box, C-term			SOCS Box, OD
D					CUL5 Box	RNA inter	N-term, C-term		SOCS Box, OD
F1				CUL5 Box	RNA inter	N-term, C-term			SOCS Box, OD
F2						N-term, RNA inter, CUL5 Box	C-term		SOCS Box, OD
G			C-term		RNA inter, CUL5 Box	N-term			SOCS Box, OD
H				RNA inter, CUL5 Box	N-term, C-term				SOCS Box, OD
01_AE						N-term, RNA inter, CUL5 Box, C-term			SOCS Box, OD
02_AG				CUL5 Box, C-term	N-term, RNA inter				SOCS Box, OD
06_cpx					CUL5 Box, C-term	N-term, RNA inter			SOCS Box, OD
07_BC							N-term, RNA inter, C-term		CUL5 Box, SOCS Box, OD
08_BC						RNA inter	N-term, C-term	CUL5 Box	SOCS Box, OD
11_cpx				C-term	CUL5 Box	N-term, RNA inter			SOCS Box, OD
12_BF						RNA inter, CUL5 Box, C-term	N-term		SOCS Box, OD
13_cpx					RNA inter	N-term, C-term	CUL5 Box		SOCS Box, OD
14_BG							CUL5 Box	N-term, RNA inter, C-term	SOCS Box, OD
15_01B						CUL5 Box	N-term, RNA inter, C-term	OD	SOCS Box
22_01A1				CUL5 Box		N-term, RNA inter, C-term			SOCS Box, OD
35_A1D							N-term, RNA inter, CUL5 Box	C-term	SOCS Box, OD
42_BF1								N-term, RNA inter	CUL5 Box, SOCS Box, OD, C-term
46_BF1						CUL5 Box	N-term, RNA inter, C-term		SOCS Box, OD
56_cpx						CUL5 Box, C-term	N-term, RNA inter		SOCS Box, OD
59_01B								N-term, RNA inter, C-term	CUL5 Box, SOCS Box, OD
63_02A6					CUL5 Box			N-term, RNA inter	SOCS Box, OD, C-term
64_BC								N-term, RNA inter, C-term	CUL5 Box, SOCS Box, OD
66_BF1						N-term, RNA inter	CUL5 Box	SOCS Box	OD
71_BF1				CUL5 Box	RNA inter, C-term	N-term			SOCS Box, OD
85_BC								N-term, RNA inter, CUL5 Box, C-term	SOCS Box, OD
89_BF1						RNA inter	N-term, CUL5 Box, C-term		SOCS Box, OD
91_cpx								N-term, RNA inter, CUL5 Box, C-term	SOCS Box, OD
103_01B							N-term, RNA inter, C-term	CUL5 Box, SOCS Box	OD
111_01C							N-term, RNA inter, C-term		CUL5 Box, SOCS Box, OD
133_A6B						SOCS Box		N-term, RNA inter, CUL5 Box, C-term	OD
137_0107							RNA inter	N-term, C-term	CUL5 Box, SOCS Box, OD
145_0755								C-term	N-term, RNA inter, CUL5 Box, SOCS Box, OD

Clade	Vpr protein - Conservation (%)						
	86.0-87.9	88.0-89.9	90.0-91.9	92.0-93.9	94.0-95.9	96.0-97.9	98.0-100
Group M		aHelix-2, C-term		N-term, aHelix-3	aHelix-1		
A1			aHelix-1, C-term	aHelix-2	N-term, aHelix-3		
A6					N-term, aHelix-1, aHelix-2, aHelix-3, C-term		
B			aHelix-2, aHelix-3, C-term	N-term, aHelix-1			
C		aHelix-2	aHelix-3, C-term	N-term	aHelix-1		
D			aHelix-2, C-term	aHelix-3	N-term, aHelix-1		
F1				aHelix-1, aHelix-2, aHelix-3, C-term	N-term		
F2			aHelix-2, aHelix-3, C-term		N-term	aHelix-1	
G			aHelix-2, C-term	N-term	aHelix-1, aHelix-3		
H			aHelix-3	N-term, aHelix-2, C-term	aHelix-1		
01_AE			aHelix-2	N-term	aHelix-1, aHelix-3, C-term		
02_AG				aHelix-2, aHelix-3, C-term	N-term, aHelix-1		
06_cpx	aHelix-2			N-term, aHelix-1, aHelix-3, C-term			
07_BC			aHelix-2	aHelix-3	N-term, C-term		aHelix-1
08_BC					aHelix-3, C-term	N-term, aHelix-2	aHelix-1
11_cpx				aHelix-2	N-term, C-term	aHelix-1, aHelix-3	
12_BF				aHelix-2	aHelix-3, C-term	N-term, aHelix-1	
13_cpx			aHelix-2, aHelix-3	C-term	N-term, aHelix-1		
14_BG					aHelix-1, aHelix-2	N-term, aHelix-3, C-term	
15_01B			aHelix-2	N-term	aHelix-1, C-term	aHelix-3	
22_01A1			aHelix-2		N-term, C-term	aHelix-3	aHelix-1
35_A1D				aHelix-3	aHelix-2, C-term	N-term, aHelix-1	
42_BF1			C-term	aHelix-3		aHelix-2	N-term, aHelix-1
46_BF1			aHelix-2		N-term, C-term	aHelix-1, aHelix-3	
56_cpx				C-term	aHelix-3	N-term, aHelix-2	aHelix-1
59_01B					aHelix-2		N-term, aHelix-1, aHelix-3, C-term
63_02A6					aHelix-2	N-term, aHelix-1, C-term	
64_BC						N-term, aHelix-1, aHelix-3, C-term	aHelix-2
66_BF1			C-term		N-term, aHelix-1, aHelix-2, aHelix-3		
71_BF1			aHelix-2, C-term	N-term, aHelix-1, aHelix-3	aHelix-3		
85_BC					aHelix-2, aHelix-3, C-term	N-term	aHelix-1
89_BF1				C-term	aHelix-3	N-term, aHelix-2	aHelix-1
91_cpx							N-term, aHelix-1, aHelix-2, aHelix-3, C-term
103_01B						N-term, aHelix-1, aHelix-3, C-term	
111_01C					N-term, aHelix-2	aHelix-1, aHelix-3, C-term	
133_A6B						aHelix-2, aHelix-3, C-term	N-term, aHelix-1
137_0107					aHelix-3	N-term, aHelix-1, C-term	aHelix-2
145_0755					aHelix-2	N-term, aHelix-1, aHelix-3, C-term	

Annotated protein motif/domain: N-term, N-terminal region; C-term, C-terminal region; RNA inter, RNA interaction motif, CUL5 Box, Cullin 5 box (HCCH zinc-binding motif); SOCS Box, suppressor of cytokine signaling box; OD, oligomerization domain.
